# Calprotectin and Sarcopenia in COPD: Biomarker, Bystander or Target?

**DOI:** 10.1002/jcsm.70283

**Published:** 2026-03-27

**Authors:** Irena Sarc, Ramon Langen, Harry Reinier Gosker, Mitja Lainscak

**Affiliations:** ^1^ University Clinic of Respiratory and Allergic Diseases Golnik Slovenia; ^2^ Faculty of Medicine University of Ljubljana Ljubljana Slovenia; ^3^ Department of Respiratory Medicine Institute NUTRIM for Nutrition and Translational Research in Metabolism, Maastricht University Medical Centre+ Maastricht the Netherlands; ^4^ General Hospital Murska Sobota Murska Sobota Slovenia

**Abbreviations**
5STS: five times sit‐to‐stand test
COPD: chronic obstructive pulmonary disease
ECOPD: exacerbation of COPD
PR: pulmonary rehabilitation
RAGE: receptor for advanced glycation end‐products
S100A8/S100A9: S100 calcium‐binding proteins A8 and A9 (forming the Calprotectin heterodimer)
TLR4: Toll‐like receptor 4

Sarcopenia is common in chronic obstructive pulmonary disease (COPD)—and it matters. Reported prevalence varies widely (up to ~50%), reflecting differences in definitions, patient populations and disease severity [1]. Risk of sarcopenia increases with age, advanced disease, comorbidity burden, ongoing smoking and systemic inflammation [2, 3]. Low muscle mass and impaired function consistently track with poorer lung function, more acute exacerbations and higher mortality of COPD patients [1, 4, 5]. Yet sarcopenia in COPD remains underdiagnosed and undertreated, underscoring the need for improved and pragmatic screening and targeted interventions [1].

While direct assessment of muscle function and mass is the golden standard for detection of sarcopenia, this requires infrastructure, expertise and time that may not readily be available at all sites. Consequently, circulating biomarkers have been explored as potential tools for sarcopenia screening [6, 7, 8]. In a recent cross‐sectional study published in the Journal, Liao et al. [9] reported that higher serum calprotectin levels were associated with sarcopenia in COPD, with good discrimination in both the development cohort (AUC 0.811) and an independent validation cohort (AUC 0.805; proposed cut‐off 78 ng/mL). Calprotectin concentrations correlated negatively with multiple muscle‐related indices, including handgrip strength, quadriceps strength and ultrasound assessment of the rectus femoris, and positively with the 5‐times sit‐to‐stand test (5STS; Figure 1). The association between calprotectin and muscle indices was reported to be independent of age and FEV_1_, although calprotectin values were higher in patients with more severe airflow limitation. Therefore, follow‐up studies, for example, with a longitudinal design are required to disentangle correlations between local disease, serum calprotectin and sarcopenia. Such longitudinal studies will enable to establish potential relationships of calprotectin levels to trajectories of muscle decline or clinical outcomes.

**FIGURE 1:**
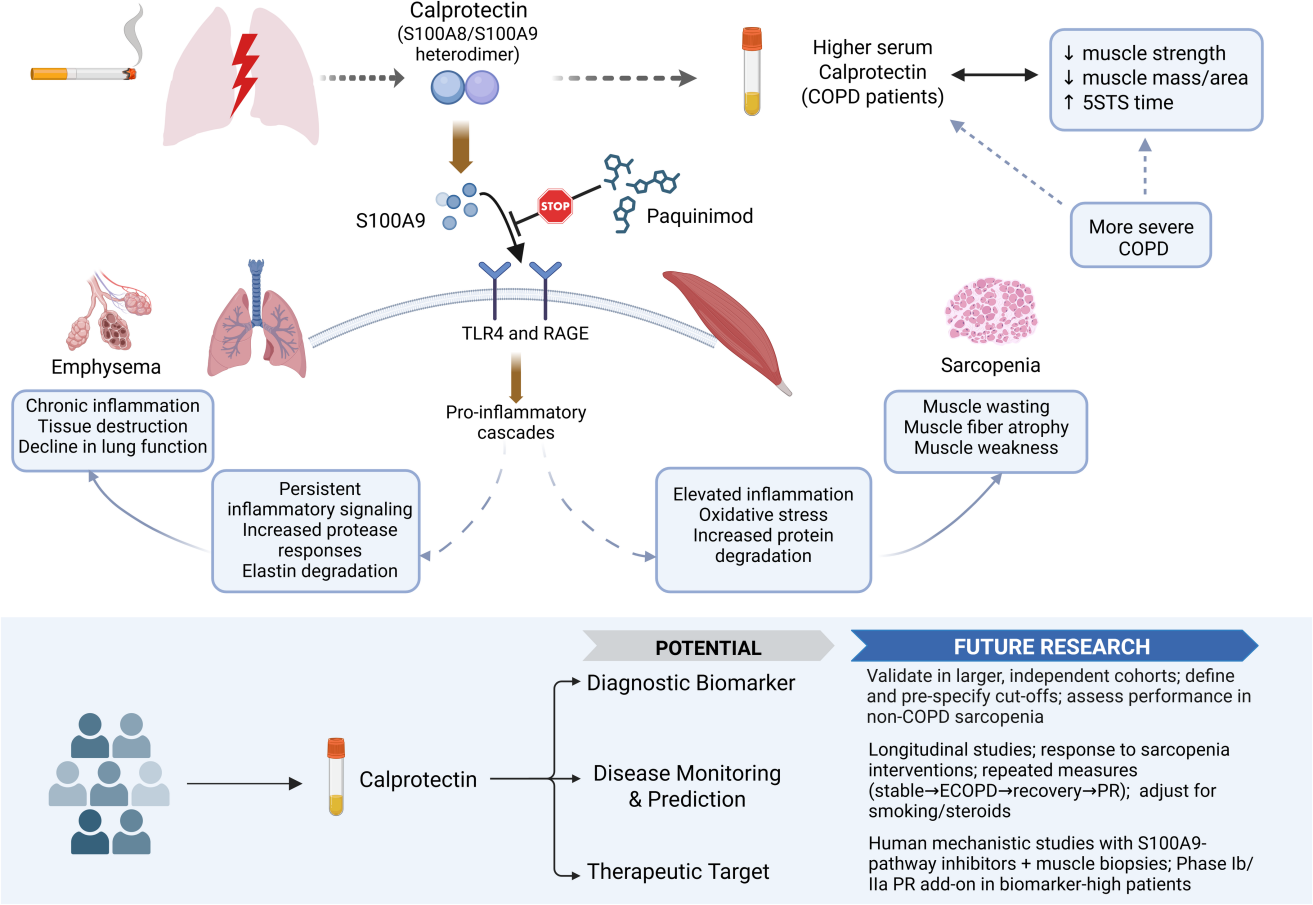
Calprotectin in chronic obstructive pulmonary disease (COPD): current status and future perspectives.

In this context it is of note that prior research suggests calprotectin predicts mortality in COPD [10], which is in line with sarcopenia as a major risk factor for mortality. Moreover, should robust evidence confirm a causal role of calprotectin in the pathogenesis of COPD‐associated muscle loss, serum calprotectin screening could be used to identify patients at increased risk of developing sarcopenia. However, reported serum calprotectin concentrations vary substantially between studies. In a previous cohort of advanced COPD, ≥70% of the patients had calprotectin ≥ 100 ng/mL [10], while in the Liao study most patients had serum levels < 100 ng/mL. Unfortunately, no healthy control group was included to provide a concurrent reference range, which is relevant as in healthy populations a wide (0.1–1.6 μg/mL) range in serum levels has been postulated [11]. Therefore, before calprotectin can be implemented clinically as a diagnostic tool, assay standardization, population‐ and age‐specific reference intervals, and prospectively validated cut‐off values will be required. Larger prospective studies will also need to clarify how corticosteroid exposure, exacerbation and current smoking status, systemic inflammatory burden, infection/colonization and comorbidities may influence circulating calprotectin, and whether these factors affect its performance as a sarcopenia biomarker (Figure 1).

Aside from a diagnostic perspective, calprotectin has also been proposed as a therapeutic target. Calprotectin is a heterodimer of the S100 calcium‐binding proteins S100A8 and S100A9 (Figure 1) and is widely regarded as a marker of innate immune activation and chronic inflammation [12]. It is highly abundant in neutrophils and monocytes and released upon cellular activation, damage or death [13]. Once extracellular, it functions as a damage‐associated molecular pattern (DAMP). Within the heterodimer, S100A8 is often described as the more cytoprotective and antimicrobial subunit, whereas S100A9 acts predominantly as a proinflammatory DAMP/alarmin that amplifies extracellular receptor signalling (e.g., via TLR4 and RAGE) and can promote tissue‐damaging inflammation (Figure 1) [12, 13]. Beyond their roles in host defence, calprotectin and its subunits have been linked to disease pathogenesis across multiple conditions. In the context of cigarette smoke (CS) exposure, increased S100A9/calprotectin has been reported in both lung compartments and circulation in murine models and patients with COPD [14, 15].

To assess the pathological contribution of calprotectin to COPD‐associated muscle dysfunction, the study by Liao et al. [9] examined pharmacological blockade using paquinimod in a mouse model of chronic CS exposure. Paquinimod is a small molecule that inhibits S100A9‐mediated calprotectin signalling and has received FDA orphan drug designation. Oral administration of paquinimod prevented CS‐associated weight loss and grip strength decline, and attenuated muscle mass loss after 3 months of CS exposure. In the gastrocnemius muscle, paquinimod prevented reductions in myofibre cross‐sectional area, suppressed activation of the ubiquitin–proteasome atrophy program (reduced Atrogin‐1 and MuRF1), and reduced local inflammation and oxidative stress. These findings should be interpreted in the context of the broader COPD muscle literature, as the development of sarcopenia features in preclinical rodent models of COPD is highly dependent on the selected model [16, 17]. Notably, CS exposure time and route (nose‐cone versus whole body) have been reported as important determinants of a skeletal muscle phenotype [16, 18], while the CS exposure of 3 months applied by Liao et al. [9] was comparatively short. Although COPD‐like lung pathology was confirmed histologically, mice were relatively young (~2 months) at initiation of the CS exposure. As mice continue to gain body mass until approximately 5 months of age, it is possible that CS affected normal tissue growth rather than inducing true muscle atrophy. As COPD typically evolves over years and mostly affects older individuals, the potential of paquinimod to prevent or reverse sarcopenia would further be strengthened by follow‐up studies using longer CS regimes in older animals. Importantly, such studies should also include female mice to rule out potential interference of sexual dimorphism described for COPD [19] and therapeutic interventions targeting muscle wasting [20].

Despite an impressive effect on muscle mass, the precise mechanism of action and in particular the primary tissue on which paquinimod exerts its effects requires further investigation. In a recent study using a similar mouse model, CS‐exposure increased pulmonary S100A9 and COPD‐like lung pathology, which was attenuated by paquinimod including a strong suppression of pulmonary inflammation [21]. These data position S100A9/calprotectin signalling upstream of CS‐induced pulmonary inflammatory and stress pathways (Figure 1). A key question therefore remains whether paquinimod muscle effects reflect direct actions on skeletal muscle or are predominantly mediated by indirect mechanisms, i.e. through attenuation of lung injury and a subsequent reduction in systemic spill‐over of inflammatory mediators that impact skeletal muscle. Addressing these issues will require specific study designs relying on preclinical COPD models that include mice with tissue‐specific deletion of S100A9 and its cognate receptors [22].

Mechanistically, the study by Liao et al. implicated activation of the ubiquitin–proteasome system (UPS) as driver of the muscle wasting. While the suppression of Atrogin‐1 and MuRF1 expression by paquinimod correlated with prevention of muscle atrophy, the relevance of these findings to the intracellular mechanisms of sarcopenia in patients with COPD is unclear as most muscle biopsy data do not support a role of UPS in the stable phase of the disease [16, 23]. Instead, evidence for UPS activation has been documented in muscle biopsies obtained from COPD patients during an acute exacerbation (ECOPD) [24], and in preclinical models of pulmonary inflammation, where muscle atrophy occurs in a UPS‐dependent manner [25]. As S100A9 serum levels are increased in ECOPD compared with stable COPD [14], ECOPD may provide a window of opportunity for therapies that prevent proteolytic activation driving acute muscle loss. In support of this notion, inhibition of S100A9 signalling was shown to attenuate sepsis‐induced muscle wasting [26].

COPD‐related muscle dysfunction extends beyond fibre atrophy and weakness and also encompasses reduced endurance linked to impaired mitochondrial capacity and a shift from oxidative towards more glycolytic fibre types, both of which contribute significantly to exercise intolerance in COPD [27, 28, 29]. These aspects were not explored in the reported mouse model, leaving unresolved whether paquinimod influenced mitochondrial function or fibre‐type composition (which in fact is known to be altered in chronically CS‐exposed mice) [30]. However, S100A8/A9 signalling has been mechanistically linked to muscle mitochondrial dysfunction in other disease contexts, including myocardial ischemia–reperfusion injury and sepsis‐induced muscle atrophy [26, 31]. This may provide a rationale for further pursuing calprotectin pathway blockade as a venue for preservation of mitochondrial capacity and fibre type composition to improve endurance‐related outcomes in COPD.

Before translation to patient care, validation in larger prospective longitudinal cohorts, mechanistic studies in human skeletal muscle, and interventional trials testing S100A9‐pathway inhibitors with appropriate clinical endpoints are warranted (Figure 1). Candidate Paquinimod has been considered safe with doses < 4.5 mg/day in early‐phase clinical trials for systemic lupus erythematosus and systemic sclerosis [32, 33]. Other candidate S100A8/A9‐blocking agents that have been tested in clinical trials are Tasquinimod (ABR‐215050) [34] and Laquinimod (ABR‐215062) [35]. Interestingly, muscle mass preserving effects of Tasquinimod have been shown preclinically, but via HDAC4 instead of S100A9 inhibition [36]. Repurposing these S100A8/A9 inhibitors as a muscle‐preserving drug in COPD would imply new risk‐benefit studies to determine its therapeutic index in this vulnerable and fragile population.

In conclusion, calprotectin could be a useful and pragmatic addition to sarcopenia screening in COPD and has therapeutical antisarcopenic potential as well. However, the utility of calprotectin/S100A9 as a clinical biomarker in COPD, and the therapeutic relevance of targeting this pathway for COPD‐associated sarcopenia needs further investigation (Figure 1).

## Funding

ML is supported by Slovenian Research and Innovation Agency (Grants Nr. P3‐0456, J3‐9292 and J3‐3076). IS is supported by Slovenian Research and Innovation Agency (Grants Nr. I0‐0062 and P3‐0360).

## Data Availability

Data sharing is not applicable to this article as no datasets were generated or analysed during the current study.

## References

[1] W. Sepúlveda‐Loyola , C. Osadnik , S. Phu , A. A. Morita , G. Duque , and V. S. Probst , “Diagnosis, Prevalence, and Clinical Impact of Sarcopenia in COPD: A Systematic Review and Meta‐Analysis,” Journal of Cachexia, Sarcopenia and Muscle 11, no. 5 (2020 Oct): 1164–1176. 32862514 10.1002/jcsm.12600 PMC7567149

[2] J. Zhou , Y. Liu , F. Yang , et al., “Risk Factors of Sarcopenia in COPD Patients: A Meta‐Analysis,” COPD 19 (2024): 1613–1622. 10.2147/COPD.S456451 PMC11246983 39011123

[3] M. K. Byun , E. N. Cho , J. Chang , C. M. Ahn , and H. J. Kim , “Sarcopenia Correlates With Systemic Inflammation in COPD,” COPD 12 (2017): 669–675. 10.2147/COPD.S130790 PMC5325093 28255238

[4] M. Lainscak , S. von Haehling , W. Doehner , et al., “Body Mass Index and Prognosis in Patients Hospitalized With Acute Exacerbation of Chronic Obstructive Pulmonary Disease,” Journal of Cachexia, Sarcopenia and Muscle 2, no. 2 (2011 Jun): 81–86. 21766053 10.1007/s13539-011-0023-9 PMC3118008

[5] M. Lainscak , T. Zupanic , D. Omersa , I. Erzen , and J. Farkas , “Prevalence of Cachexia and Outcomes in Patients With Chronic Diseases: A National Database Analysis of 5 484 103 Hospitalisations,” Journal of Cachexia, Sarcopenia and Muscle 16, no. 1 (2025): e13688. 39831326 10.1002/jcsm.13688 PMC11744301

[6] P. Aparicio , D. Navarrete‐Villanueva , A. Gómez‐Cabello , et al., “Proteomic Profiling of Human Plasma Extracellular Vesicles Identifies PF4 and C1R as Novel Biomarker in Sarcopenia,” Journal of Cachexia, Sarcopenia and Muscle 15, no. 5 (2024 Oct): 1883–1897. 39009419 10.1002/jcsm.13539 PMC11446689

[7] K. Hirai , A. Tanaka , T. Homma , et al., “Serum Creatinine/Cystatin C Ratio as a Surrogate Marker for Sarcopenia in Patients With Chronic Obstructive Pulmonary Disease,” Clinical Nutrition 40, no. 3 (2021): 1274–1280. 32863062 10.1016/j.clnu.2020.08.010

[8] R. Qaisar , A. Karim , T. Muhammad , I. Shah , and J. Khan , “Prediction of Sarcopenia Using a Battery of Circulating Biomarkers,” Scientific Reports 11, no. 1 (2021): 8632. 33883602 10.1038/s41598-021-87974-6 PMC8060253

[9] L. Liao , J. Li , W. Xu , et al., “Calprotectin Is a Circulating Biomarker and Potential Therapeutic Target for Sarcopeni in Chronic Obstructive Pulmonary Disease,” Journal of Cachexia, Sarcopenia and Muscle 17, no. 1 (2026): e70196. 41582634 10.1002/jcsm.70196 PMC12833497

[10] D. B. Holmgaard , L. H. Mygind , I. Titlestad , et al., “Calprotectin—A Marker of Mortality in COPD? Results From a Prospective Cohort Study,” COPD: Journal of Chronic Obstructive Pulmonary Disease 10, no. 5 (2013): 581–587. 23844942 10.3109/15412555.2013.781580

[11] M. Jarlborg , D. S. Courvoisier , C. Lamacchia , et al., “Serum Calprotectin: A Promising Biomarker in Rheumatoid Arthritis and Axial Spondyloarthritis,” Arthritis Research & Therapy 22, no. 1 (2020): 105. 32375861 10.1186/s13075-020-02190-3 PMC7201559

[12] O. S. Kotsiou , D. Papagiannis , R. Papadopoulou , and K. I. Gourgoulianis , “Calprotectin in Lung Diseases,” International Journal of Molecular Sciences 22, no. 4 (2021): 1706. 33567747 10.3390/ijms22041706 PMC7915440

[13] S. Wang , R. Song , Z. Wang , Z. Jing , S. Wang , and J. Ma , “S100A8/A9 in Inflammation,” Frontiers in Immunology 9, no. 11 (2018): 1298. 29942307 10.3389/fimmu.2018.01298 PMC6004386

[14] S. D. Pouwels , M. C. Nawijn , E. Bathoorn , et al., “Increased Serum Levels of LL37, HMGB1 and S100A9 During Exacerbation in COPD Patients,” European Respiratory Journal 45, no. 5 (2015): 1482–1485. 25931489 10.1183/09031936.00158414

[15] R. F. Foronjy , P. O. Ochieng , M. A. Salathe , et al., “Protein Tyrosine Phosphatase 1B Negatively Regulates S100A9‐Mediated Lung Damage During Respiratory Syncytial Virus Exacerbations,” Mucosal Immunology 9, no. 5 (2016 Sep): 1317–1329. 26813343 10.1038/mi.2015.138 PMC4963308

[16] P. Henrot , I. Dupin , P. Schilfarth , et al., “Main Pathogenic Mechanisms and Recent Advances in COPD Peripheral Skeletal Muscle Wasting,” IJMS. 24, no. 7 (2023): 6454. 37047427 10.3390/ijms24076454 PMC10095391

[17] R. Vlahos and S. Bozinovski , “Preclinical Murine Models of Chronic Obstructive Pulmonary Disease,” European Journal of Pharmacology 759, no. 15 (2015): 265–271. 25818750 10.1016/j.ejphar.2015.03.029

[18] M. Rinaldi , K. Maes , S. De Vleeschauwer , et al., “Long‐Term Nose‐Only Cigarette Smoke Exposure Induces Emphysema and Mild Skeletal Muscle Dysfunction in Mice,” Disease Models & Mechanisms 5 (2012): 333–341. 22279084 10.1242/dmm.008508 PMC3339827

[19] A. Tam , J. H. T. Bates , A. Churg , J. L. Wright , S. F. P. Man , and D. D. Sin , “Sex‐Related Differences in Pulmonary Function Following 6 Months of Cigarette Exposure: Implications for Sexual Dimorphism in Mild COPD,” PLoS ONE 11, no. 10 (2016): e0164835. 27788167 10.1371/journal.pone.0164835 PMC5082824

[20] S. Tsitkanou , F. Morena da Silva , A. R. Cabrera , et al., “Mitochondrial Antioxidant SkQ1 Attenuates C26 Cancer‐Induced Muscle Wasting in Males and Improves Muscle Contractility in Female Tumor‐Bearing Mice,” American Journal of Physiology‐Cell Physiology 327, no. 5 (2024): C1308–C1322. 39344417 10.1152/ajpcell.00497.2024 PMC11559642

[21] C. Railwah , A. Lora , K. Zahid , et al., “Cigarette Smoke Induction of S100A9 Contributes to Chronic Obstructive Pulmonary Disease,” American Journal of Physiology. Lung Cellular and Molecular Physiology 319, no. 6 (2020): L1021–L1035. 32964723 10.1152/ajplung.00207.2020 PMC7938777

[22] S. Huo , M. Wang , M. Du , et al., “Macrophage‐Derived S100A9 Promotes Diabetic Cardiomyopathy by Disturbing Mitochondrial Quality Control via STAT3 Activation,” International Journal of Biological Sciences 21, no. 7 (2025): 3061–3080. 40384874 10.7150/ijbs.111128 PMC12080395

[23] A. E. M. Kneppers , R. C. J. Langen , H. R. Gosker , et al., “Increased Myogenic and Protein Turnover Signaling in Skeletal Muscle of Chronic Obstructive Pulmonary Disease Patients With Sarcopenia,” Journal of the American Medical Directors Association 18, no. 7 (2017): 637.e1–637.e11. 10.1016/j.jamda.2017.04.016 28578881

[24] T. Crul , D. Testelmans , M. A. Spruit , et al., “Gene Expression Profiling in Vastus Lateralis Muscle During an Acute Exacerbation of COPD,” Cellular Physiology and Biochemistry 25, no. 4–5 (2010): 491–500. 20332630 10.1159/000303054

[25] J. J. M. Ceelen , A. M. W. J. Schols , N. G. M. Thielen , et al., “Pulmonary Inflammation‐Induced Loss and Subsequent Recovery of Skeletal Muscle Mass Require Functional Poly‐Ubiquitin Conjugation,” Respiratory Research 19, no. 1 (2018): 80. 29720191 10.1186/s12931-018-0753-8 PMC5932886

[26] D. Huang , Y. Li , Y. Guo , et al., “Elevated Levels of S100A8 and S100A9 Exacerbate Muscle Mitochondrial Fragmentation in Sepsis‐Induced Muscle Atrophy,” Communications Biology 8, no. 1 (2025): 338. 40021770 10.1038/s42003-025-07654-3 PMC11871300

[27] H. R. Gosker , M. K. C. Hesselink , H. Duimel , K. A. Ward , and A. M. W. J. Schols , “Reduced Mitochondrial Density in the Vastus Lateralis Muscle of Patients With COPD,” European Respiratory Journal 30, no. 1 (2007 Jul): 73–79. 17428811 10.1183/09031936.00146906

[28] H. R. Gosker , H. Van Mameren , P. J. Van Dijk , et al., “Skeletal Muscle Fibre‐Type Shifting and Metabolic Profile in Patients With Chronic Obstructive Pulmonary Disease,” European Respiratory Journal 19, no. 4 (2002): 617–625. 11998989 10.1183/09031936.02.00762001

[29] P. A. Leermakers , A. M. W. J. Schols , A. E. M. Kneppers , et al., “Molecular Signalling Towards Mitochondrial Breakdown Is Enhanced in Skeletal Muscle of Patients With Chronic Obstructive Pulmonary Disease (COPD),” Scientific Reports 8, no. 1 (2018): 15007. 30302028 10.1038/s41598-018-33471-2 PMC6177478

[30] H. R. Gosker , R. C. J. Langen , K. R. Bracke , et al., “Extrapulmonary Manifestations of Chronic Obstructive Pulmonary Disease in a Mouse Model of Chronic Cigarette Smoke Exposure,” American Journal of Respiratory Cell and Molecular Biology 40, no. 6 (2009 Jun): 710–716. 18988919 10.1165/rcmb.2008-0312OC

[31] Y. Li , B. Chen , X. Yang , et al., “S100a8/a9 Signaling Causes Mitochondrial Dysfunction and Cardiomyocyte Death in Response to Ischemic/Reperfusion Injury,” Circulation 140, no. 9 (2019): 751–764. 31220942 10.1161/CIRCULATIONAHA.118.039262

[32] R. Hesselstrand , J. H. W. Distler , G. Riemekasten , et al., “An Open‐Label Study to Evaluate Biomarkers and Safety in Systemic Sclerosis Patients Treated With Paquinimod,” Arthritis Research & Therapy 23, no. 1 (2021): 204. 34330322 10.1186/s13075-021-02573-0 PMC8325221

[33] A. A. Bengtsson , G. Sturfelt , C. Lood , et al., “Pharmacokinetics, Tolerability, and Preliminary Efficacy of Paquinimod (ABR‐215757), a New Quinoline‐3‐Carboxamide Derivative: Studies in Lupus‐Prone Mice and a Multicenter, Randomized, Double‐Blind, Placebo‐Controlled, Repeat‐Dose, Dose‐Ranging Study in Patients With Systemic Lupus Erythematosus,” Arthritis and Rheumatism 64, no. 5 (2012 May): 1579–1588. 22131101 10.1002/art.33493

[34] B. Escudier , S. Faivre , E. Van Cutsem , et al., “A Phase II Multicentre, Open‐Label, Proof‐of‐Concept Study of Tasquinimod in Hepatocellular, Ovarian, Renal Cell, and Gastric Cancers,” Targeted Oncology 12, no. 5 (2017): 655–661. 28798986 10.1007/s11523-017-0525-2

[35] S. Constantinescu and C. Constantinescu , “Laquinimod (ABR‐215062) for the Treatment of Relapsing Multiple Sclerosis,” Expert Review of Clinical Pharmacology 9, no. 1 (2016): 49–57. 26536299 10.1586/17512433.2016.1108189

[36] D. Liang , D. Wang , X. Zheng , et al., “Aerobic Plus Resistance Exercise Attenuates Skeletal Muscle Atrophy Induced by Dexamethasone Through the HDAC4/FoxO3a Pathway,” Cellular Signalling 127 (2025): 111581. 39732306 10.1016/j.cellsig.2024.111581

